# Robotic Living Donor Hysterectomy for Uterus Transplantation: An Update on Donor and Recipient Outcomes

**DOI:** 10.3390/jcm13144186

**Published:** 2024-07-17

**Authors:** Andrew Jacques, Giuliano Testa, Liza Johannesson

**Affiliations:** Division of Abdominal Transplantation, Annette C. and Harold C. Simmons Transplant Institute, Baylor University Medical Center, 3410 Worth St, Ste 950, Dallas, TX 75246, USA; andrew.jacques@bswhealth.org (A.J.); giuliano.testa@bswhealth.org (G.T.)

**Keywords:** uterus, transplantation, infertility, live donation, robotic

## Abstract

**Background/Objectives:** Uterus transplantation (UTx) has evolved into a clinical reality for women with absolute uterine infertility. The international experience with UTx has predominantly utilized living donor grafts—and strategies to minimize harm to donors remain paramount. Robotic living donor hysterectomy represents a minimally invasive approach to facilitate rapid donor recovery, improve pelvic visualization and operative access, and maintain UTx recipient outcomes. The aim of this study is to describe donor, recipient, graft, and pregnancy outcomes after adoption of a robotic living donor hysterectomy program. **Methods:** The Dallas UtErus Transplant Study (DUETS) incorporated a robotic living donor hysterectomy operative protocol, including transvaginal extraction, from April 2019. Prospectively collected data were analyzed, and a case series presented, to describe donor intra- and post-operative outcomes and recipient intra-operative outcomes, graft viability, established pregnancies, and live births. Early cases were compared to later cases to better describe the learning curve associated with the technique. **Results:** Sixteen robotic living donor hysterectomies were performed with 100% graft viability after implantation demonstrated by myometrial flow and onset of menses. Early experience (eight cases) demonstrated two cases of ureteric injury. Later experience (eight cases) demonstrated a reduction in operative time (11 h 10 min vs. 6 h 38 min), with no ureteric injuries and a reduction in major operative morbidity from 25% to 12.5% (Clavien–Dindo grade ≥3). At the time of reporting, nine successful live births have occurred, with six ongoing pregnancies. **Conclusions:** Robotic living donor hysterectomy represents a safe approach to minimize donor harm without compromising UTx recipient, graft, and pregnancy-related outcomes. A learning curve is demonstrated with the adoption of the novel technique—with particularly care required to prevent ureteric injuries, and ongoing vigilance and reporting necessary given the small case numbers of robotic living donor hysterectomy reported internationally.

## 1. Introduction

From experimental beginnings, uterus transplantation (UTx) has now become a clinical reality for women with absolute uterine factor infertility (AUFI). From the success reported to date, the majority of live births have been the product of grafts procured through living donation [1,2]. While deceased donor UTx avoids the potential harm to the donor, the opportunity to both complete a comprehensive work-up of the donor, and to schedule complex, multi-disciplinary surgery means that living donation will remain a cornerstone of UTx for the foreseeable future. Consequently, the desire to minimize the risk of harm to the donor remains paramount.

Early experiences with living donor hysterectomy were via an open approach, with subsequent reports describing various laparoscopic or hybrid techniques [3,4,5]. The motivation for pursuing more minimally invasive approaches is the desire to minimize pain and hasten recovery for the donor without compromising the safety of the surgery. These goals have previously been mirrored in the transition to minimally invasive live donor nephrectomy for transplantation [6,7], as well as in hysterectomies performed outside the transplantation context [8,9].

Unique challenges, however, exist in living donor hysterectomy that are not present when performing simple hysterectomy for benign disease. Preservation of the vascular pedicle is of particular importance for the success of the subsequent implantation—requiring (1) careful dissection of the ureters as they are crossed by the uterine artery, and (2) preservation of the uterine veins. Given these critical steps, robotic surgery has the potential to provide advantages in a narrow operative field and to improve visualization when compared to open surgery.

Our group has previously published on our early robotic experience after initially utilizing an open approach [10,11]. From the five cases reported, while operative time was increased with a robotic approach, there was a reduction in the estimated blood loss and length of hospital stay. While the complication rate between open and robotic cases was not demonstrably different, ureteric injuries were identified in the early post-operative phase in living donors following the robotic approach. Consequently, ongoing vigilance and reporting of both the risks and benefits of this more minimally invasive approach is warranted as case experience continues to grow. The aim of this study is to report the outcomes and complications for both the living donor and recipient following adoption of a robotic approach to living donor hysterectomy.

## 2. Materials and Methods

### 2.1. Overview

The Dallas UtErus Transplant Study (DUETS) commenced as an experimental clinical trial following institutional review board approval (NCT02656550). After initially commencing in November 2015, living donor hysterectomies were performed as robot-assisted procedures from April 2019. Donors were all self-referred and provided written informed consent to proceed with robotic living donor hysterectomy following counselling about the team experience with the procedure. The donors all underwent comprehensive evaluation, including medical and psychological assessment for suitability to donate. All donors explicitly stated their desire for no further children and had a history of previous successful pregnancy. For recipients, in vitro fertilization (IVF) is mandatory for live birth following UTx as intercourse will not lead to pregnancy following division of the oviducts. The first IVF cycles were performed prior to UTx to avoid complications that may arise secondary to changes in pelvic anatomy, complications of immunosuppression, and to ensure sufficient embryos can be frozen to justify subsequent transplantation.

### 2.2. Donor Surgery

The robotic living donor hysterectomies were all performed using the da Vinci Xi robotic system. Surgery was performed with the patient in Trendelenburg position (15 degrees all cases), utilizing CO_2_ pneumoperitoneum (<12 mmHg), with a four robotic arm arrangement. Ureteric stents placed bilaterally were standard of care (donors only), and indocyanine green was injected retrograde to facilitate ureter identification during dissection. Retraction of the uterus was performed with a uterine manipulator (Rumi II Koh-Efficient Uterine Manipulator; CooperSurgical, Trumbull, CT, USA).

The operative steps performed are as follows: (1) lateral division of the round ligaments to facilitate entering the retro-peritoneum; (2) further dissection to open the pararectal and paravesical spaces; (3) internal iliac artery identification and distal dissection to the level of the obliterated umbilical artery; (4) separation of the bladder from the front wall of the uterus, cervix, and upper vagina; (5) careful dissection of the ureters down to their bladder insertion, with specific attention to the uterine pedicle superiorly; (6) exposure of the ureteric tunnel; (7) posterior vaginal dissection away from the rectum; (8) identification and ligation of tributaries to the internal iliac veins, excluding the uterine veins; (9) utero-ovarian and fallopian tube dissection; (10) vaginal transection; (11) transection of vascular inflow and outflow (utero-ovarian veins, uterine arteries, and uterine veins); (12) graft extraction via the vagina utilizing a EndoCatch^TM^ retrieval system; (13) closure of vaginal cuff.

### 2.3. Recipient Surgery

UTx proceeds immediately after the robotic living donor hysterectomy is completed to minimize cold ischemic time, but only following the initial back-table assessment and cold preservation flush (Custodial HTK, Essential Pharmaceuticals, Durham, NC, USA). The graft is preserved in preservation solution at approximately 0–4 °C to minimize metabolic activity prior to revascularization. Implantation was performed via an open approach with a lower midline laparotomy. Open surgery remains the preferred approach for the implantation, in order to meticulously complete the vascular reconstruction and to minimize warm ischemic time prior to reperfusion. Arterial reconstruction to establish inflow is completed with anastomoses between bilateral uterine arteries with their associated portion of internal iliac artery to the recipient’s external iliac arteries. The recipient’s native uterine vasculature is not utilized for implantation (and may be congenitally absent in the context of MRKH). Venous reconstruction to establish outflow is completed anastomoses to the recipient’s external iliac vein utilizing either (1) utero-ovarian (superior uterine) veins, (2) bilateral uterine (inferior uterine) veins, or (3) a combination of the two veins.

### 2.4. Immunosuppression and Rejection Surveillance

All UTx recipients received anti-thymocyte globulin with methylprednisolone as induction immunosuppression (4.5 mg/kg and 500–1000 mg, respectively). Immunosuppression maintenance was with a combination of tacrolimus and either azathioprine or sirolimus. Level monitoring was performed every 1–2 weeks with a tacrolimus goal of 9–11 ng/mL from transplant to 3 months, with a 4–8 ng/mL goal beyond 3 months. Protocol cervical biopsies were performed for rejection surveillance every 2 weeks for 3 months, and then monthly until the time of embryo transfer. The UTx rejection grading scale was utilized for biopsy reporting [12]. Immunosuppression was continued throughout pregnancy, and only ceased if/when graft hysterectomy was completed.

### 2.5. Graft Monitoring and Pregnancy

Recipient early graft function was defined as established myometrial blood flow on ultrasonography, on day 1 and day 5 post-operatively, assessment of day 5 cervical biopsy, and the subsequent establishment of menstrual bleeding. Prenatal obstetrics care was provided via 2–3 weekly assessments following single embryo transfer only. All deliveries were via cesarean section, with consideration of graft hysterectomy after consultation between the UTx recipient and the physician team.

### 2.6. Donor Operative and Post-Operative Outcomes

Intra-operative donor outcome measures included operative time, estimated blood loss, vessels successfully isolated and retrieved, and unplanned conversion to open surgery. Post-operative outcome measures included hospital length of stay and complications graded as per the Clavien–Dindo classification [13].

### 2.7. Recipient and Pregnancy Outcomes

Intra-operative recipient outcomes measures included operative time, estimated blood loss, vessels successfully implanted, and the associated implantation time. Graft viability and pregnancy outcomes measures included establishment of myometrial flow, commencement of menses, embryo transfer, established pregnancy, and successful live birth.

## 3. Results

### 3.1. Donor and Recipient Characteristics

Sixteen living donor robotic hysterectomies were performed from April 2019 to May 2024. The donor characteristics are summarized in Table 1. The mean donor age and body mass index (BMI) were 36 years and 23 kg/m^2^, respectively. All donors had previously had successful vaginal deliveries (range 1–4), and three donors had undergone a previous cesarean section (one only for all donors). The mean UTx recipient age was 33 years, with all recipients undergoing UTx for AUFI secondary to a congenital uterine absence (Mayer–Rokitansky–Küster–Hauser syndrome).

### 3.2. Donor Intra-Operative and Post-Operative Outcomes

The donor intra- and post-operative outcomes are summarized in Table 2. Comparing the first eight robotic living donor cases to the subsequent eight cases, the total operating time was 11 h 10 min versus 6 h 38 min, and estimated blood loss 145 mL vs. 75 mL. No cases were converted to open. The first eight cases successful retrieved two veins (3/8 cases), three veins (4/8 cases), and four veins (1/8 cases), compared to three veins (2/8 cases) and four veins (6/8 cases) in the latter group. The mean length of stay for both groups was 3 days. The rate of major morbidity (Clavien–Dindo grade 3 or more) occurred in 25% of early cases (2/8), compared to 12.5% in the latter group (1/8).

### 3.3. Recipient Intra-Operative, Graft, and Pregnancy Outcomes

The intra-operative outcomes are summarized in Table 1. The mean operative time, estimated blood loss, and implantation time were 4 h 34 min, 275 mL, and 72 min, respectively. All UTx performed following robotic living donor hysterectomy were technically successful—as defined by the establishment of blood flow on ultrasound and the commencement of menses. The graft and pregnancy-related outcomes are summarized in Table 3. Onset of menses occurred between 12 and 83 days, and first embryo transfer between 3–8 months. To date, nine live births have occurred (gestational age range 32 + 5 to 38 + 1 weeks), with six established pregnancies ongoing. One hypospadia and one genetic visual impairment has been identified, with all newborns appropriate for their gestational age, and no issues identified with subsequent development.

Hospital length of stay was 5–6 days for all recipients, excluding one case with a 32-day length of stay (RDR8). The case represents a major morbidity, with sepsis and ileus secondary to a rectal injury.

## 4. Discussion

The case series reported represents the largest, single-center series of robotic living donor hysterectomy in UTx. Overall, the described results demonstrate the success of the approach in both minimizing harm to the donor while maintaining successful recipient, graft, and pregnancy outcomes. The robotic approach with transvaginal extraction has resulted in a donor median length of stay of three days with minimal blood loss, all while providing a living donor uterus graft that has resulted in a 100% technical success rate since adoption of the approach. With increasing institutional experience, this success has occurred with a simultaneous reduction in the time for donor surgery, and an increase in the number of uterus draining veins procured per procedure.

Our early experience with robotic living donor hysterectomy and transvaginal extraction has previously been reported [10,11]. While the initial operative times were longer with the robotic platform when compared to an open approach, this report confirms our expectations that the operative time would reduce with increasing experience. The times reported, however, remain comparable or even shorter than other reported open living donor hysterectomy case series [14,15]. The use of transvaginal extraction remains our standard practice, with no adverse events or harm to the graft identified when utilizing the approach. To date, there have also been no cases converted to open surgery for patient safety or an inability to progress robotically. The major concern identified from our early experience was the rate of ureteric complications and the associated need for further intervention. Fortunately, through critical review of selected cases and institutional practices, paired with an ever-growing clinical and operative experience, we have not had any further ureteric injuries.

The dissection and preservation of the donor ureters and uterine veins represent the most surgically challenging aspects of living donor hysterectomy. The technical challenge with ureteric dissection pertains to the relationship between the ureter and the uterine vascular pedicle. Given the need to preserve length of the uterine pedicle to facilitate subsequent implantation, there is a need to dissect the ureters away from the overlying uterine artery. The ureters are most at risk of injury at this stage of procurement. While our group had no injuries during the initial open hysterectomy phase of our UTx program, other groups reported injuries following open surgery [3,14,15]. In earlier robotic case series, ureteric injuries either did not occur or were not reported [5,16]. The first of our ureteric complications involved a ureteric blood clot that required stenting and resolved without further intervention after the stent was removed at 8 weeks. This case may not reflect a true ureteric injury secondary to robotic dissection, but more likely an event after elective peri-operative stent insertion for the donor surgery. Regardless, the case requires discussion and identification as a potential source of donor harm. The second case, however, was more concerning with bilateral ureteric injuries attributed to thermal injury. Following review of the case other ureteric injuries were identified by other surgical services operating in the pelvis—with the root cause attributed to a thermal setting discrepancy with the device. These two cases highlight to risk of ureteric concerns when performing living donor hysterectomy, but fortunately no further events were identified in the later donor cohort.

After ureteric dissection, the second predominant technical challenge is dissection of the uterine veins and their preservation for subsequent implantation. The robotic platform potentially provides a significant advantage with regards to the outflow vessels, as the visualization of the smaller tributaries around the ureter and uterus is enhanced within the narrow space. The success of the robotic approach is demonstrated by the increasing number of veins procured with increasing experience, but also the ability to keep blood loss to a minimum. Inadvertent injury to these small veins can lead to significant bleeding and donor complications, all minimized with use of the robotic platform. Graft loss has also been reported in the published literature at approximately 24–27% [1,2], commonly a consequence of vascular / thrombotic events. The ability of the robotic approach to safely and delicately dissect and isolate critical vascular pedicles may be a contributor to the 0% graft loss reported in our series. Given the size and fragility of the procured veins for implantation, it is the opinion of the authors that the quality and number of veins procured allows for a greater range of implantation options to minimize the risk of vascular complications. While variability exists between donors, we feel the general number and quality of veins procured are influenced by the skill and experience of the donor surgeon.

The pregnancy outcomes reported are favorable when compared to that reported internationally. While many of our UTx recipients remain on their journey to parenthood following robotic living donor hysterectomy, the nine live births to date demonstrates the success of the technique. To further minimize the risk of harm during UTx (either psychological distress or the risks associated with long-term immunosuppression), our protocol has also reduced the time from UTx to embryo transfer from the 12 months employed in early trials [17,18,19]. From 13 patients having undergone embryo transfer, 11 occurred between 3–6 months to facilitate early pregnancy, live birth, and subsequent graft hysterectomy with cessation of immunosuppression. While unrelated to robotic donor procurement, RDR8 had complications secondary to a rectal injury that occurred during implantation. The case highlights the risks of major pelvic surgery, and the ongoing need for vigilance and open reporting of outcomes.

Significant limitations persist within our report. The case series remains small, from a single center, and is under-powered to make more statistically supported conclusions. As case numbers and participating institutions grow, international reporting and ongoing vigilance for donor and recipient outcomes following robotic living donor hysterectomy remain paramount. While the results described are highly encouraging, problems less frequent and yet to be recognized have the potential to arise. To optimize outcomes, we would advocate that institutions wishing to embrace UTx ensure adequate case volume and internal case review while “on the learning curve” to maximize donor and recipient outcomes. We would also advocate that new adopters of the technique collaborate with more established centers at least initially—and ensure a robust multi-disciplinary engagement within their own institutions.

## 5. Conclusions

Overall, the results of this single-center case series are incredibly encouraging. From the results presented, robotic living donor hysterectomy appears safe for the donor, recipient, and subsequent pregnancy, while minimizing donor harm by enhancing recovery and minimizing blood loss when compared to an open approach. While the early experience with the technique raised concern for potential ureteric injuries, with increasing case load and institutional understanding both operative time and rate of major complications substantially reduced, with no reported subsequent graft loss. While ongoing reporting and international collaboration remains of utmost importance, the successful live births to date demonstrate the success of the approach and emphasize the enthusiasm for robotic living donor hysterectomy moving forward.

## Figures and Tables

**Table 1:** Living donor characteristics, intra-operative, and post-operative outcomes following robotic living donor hysterectomy.

Donor	Demographic			Intra-Operative			Post-Operative	
Case	Age (yrs)	BMI (kg/m^2^)	Vaginal Deliveries	Time (h:min)	EBL (mL)	Vessels Retrieved	LOS (Days)	Complication (Clavien–Dindo Grade ≥3)
RLD1	30	19	1	9:25	150	(L) SUV; (R) SUV	4	-
RLD2	31	23	3	10:48	100	(L) SUV; (R) SUV	6	Ureteric blood clot requiring stent placement
RLD3	38	24	2	12:10	220	(L) SUV; (R) SUV IUV	3	Bilateral ureteric injury requiring reimplantation
RLD4	32	18	2	9:27	20	(L) SUV; (R) SUV IUV	4	-
RLD5	38	27	2	12:03	100	(L) SUV; (R) SUV	3	-
RLD6	31	24	4	11:04	275	(L) SUV; (R) SUV IUV	3	-
RLD7	36	28	3	12:09	100	(L) SUV IUV; (R) SUV IUV	4	-
RLD8	42	24	1	12:13	200	(L) SUV; (R) SUV IUV	3	-
RLD 1-8 (mean)	34	23		11:10	145			
RLD9	34	24	3	9:21	150	(L) SUV IUV; (R) SUV IUV	4	-
RLD10	40	22	4	6:39	50	(L) SUV IUV; (R) SUV IUV	3	-
RLD11	41	27	1	5:57	50	(L) SUV; (R) SUV IUV	3	-
RLD12	42	23	1	7:05	50	(L) SUV IUV; (R) SUV IUV	3	-
RLD13	33	23	3	6:24	100	(L) SUV IUV; (R) SUV IUV	3	-
RLD14	32	21	3	5:46	50	(L) SUV IUV; (R) SUV	3	Vaginal dehiscence
RLD15	43	24	2	6:02	50	(L) SUV IUV; (R) SUV IUV	3	-
RLD16	34	25	4	6:50	100	(L) SUV IUV; (R) SUV IUV	3	-
RLD 1-8 (mean)	33	24		6:38	75			

BMI: body mass index; RLD: robotic living donor; EBL: estimated blood loss; LOS: length of stay; SUV: superior uterine vein; IUV: inferior uterine vein.

**Table 2:** Uterus transplant recipient characteristics and intra-operative outcomes following robotic living donor hysterectomy.

Case	Age (yrs)	Time (h:min)	EBL (mL)	Vessels Implanted	Implantation Time (min)	LOS (Days)
RDR1	31	4:21	200	(L) SUV; (R) SUV	60	6
RDR2	34	4:32	500	(L) SUV; R) SUV IUV	63	6
RDR3	33	4:42	300	(L) SUV; R) SUV IUV	80	6
RDR4	34	4:58	750	(L) SUV; (R) SUV	49	6
RDR5	31	4:31	50	(L) SUV; (R) SUV	65	5
RDR6	35	4:46	300	(L) IUV; (R) SUV	74	5
RDR7	43	4:43	200	(L) IUV; (R) IUV	72	6
RDR8	36	5:57	200	(L) IUV; (R) SUV	54	32
RDR9	30	4:20	300	(L) SUV IUV; (R) SUV IUV	82	5
RDR10	38	4:15	100	(L) SUV IUV; (R) IUV	87	5
RDR11	25	4:07	100	(L) SUV; (R) IUV	65	6
RDR12	33	4:06	200	(L) SUV IUV; (R) SUV IUV	74	6
RDR13	25	4:03	200	(L) SUV; (R) SUV	83	5
RDR14	25	4:30	400	(L) SUV; (R) SUV	69	6
RDR15	39	4:08	400	(L) SUV IUV; (R) IUV	87	5
RDR16	28	5:03	200	(L) SUV IUV; (R) SUV IUV	89	6

EBL: estimated blood loss; LOS: length of stay; RDR: robotic donor recipient; SUV: superior uterine vein; IUV: inferior uterine vein.

**Table 3:** Graft and pregnancy outcomes following robotic living donor hysterectomy.

Case	Onset Menses (Days)	Time to First ET (Months)	Miscarriage	Ongoing Pregnancy	Deliveries	Time to Delivery (Months)	Gestational Age	Birth Weight (Grams)	Delivery Indication
RDR1	22	6		no	1	14	37	3025	elective
RDR2	31	4	1 (6 wks)	yes	1	47	38+1	3510	elective
RDR3	22	4		no	1	11	37	2350	PTL
RDR4	27	8		no	1	16	35 + 6	2325	PROM
RDR5	42	5		no	1	13	35.6		PTL
RDR6	12	5		no	2	13	33 + 3, 37 + 1	2688, 2650	PROM (1), elective (2)
RDR7	31	3		no	1	17	32 + 5	1632	PROM
RDR8	24	7		yes	0	TBD			
RDR9	24	6		no	1	14	37		elective
RDR10	32	6	1 (13 wks)	no	0	TBD			
RDR11	83	5		yes	0	TBD			
RDR12	40	4		yes	0	TBD			
RDR13	33	3		yes	0	TBD			
RDR14	37	TBD		yes	0	TBD			
RDR15	34	TBD		no	0	TBD			
RDR16	30	TBD		no	0	TBD			

ET: embryo transfer; RDR: robotic donor recipient; TBD: to be determined; PROM = premature rupture of membranes; PTL = pre-term labor.

## Data Availability

Data are contained within the article.
